# High-Grade Endometrial Stromal Sarcoma: Molecular Alterations and Potential Immunotherapeutic Strategies

**DOI:** 10.3389/fimmu.2022.837004

**Published:** 2022-02-15

**Authors:** Youngah Kim, Dohyang Kim, Woo Jung Sung, Jaewoo Hong

**Affiliations:** ^1^ Department of Physiology, Daegu Catholic University School of Medicine, Daegu, South Korea; ^2^ Department of Pathology, Daegu Catholic University School of Medicine, Daegu, South Korea

**Keywords:** BCOR sarcoma, rare cancer, stromal sarcoma, tumor microenvironment, inflammation

## Abstract

Endometrial stromal tumor (EST) is an uncommon and unusual mesenchymal tumor of the uterus characterized by multicolored histopathological, immunohistochemical, and molecular features. The morphology of ESTs is similar to normal endometrial stromal cells during the proliferative phase of the menstrual cycle. ESTs were first classified into benign and malignant based on the number of mitotic cells. However, recently WHO has divided ESTs into four categories: endometrial stromal nodules (ESN), undifferentiated uterine sarcoma (UUS), low-grade endometrial stromal sarcoma (LG-ESS), and high-grade endometrial stromal sarcoma (HG-ESS). HG-ESS is the most malignant of these categories, with poor clinical outcomes compared to other types. With advances in molecular biology, ESTs have been further classified with morphological identification. ESTs, including HG-ESS, is a relatively rare type of cancer, and the therapeutics are not being developed compared to other cancers. However, considering the tumor microenvironment of usual stromal cancers, the advance of immunotherapy shows auspicious outcomes reported in many different stromal tumors and non-identified uterine cancers. These studies show the high possibility of successful immunotherapy in HG-ESS patients in the future. In this review, we are discussing the background of ESTs and the BCOR and the development of HG-ESS by mutations of BCOR or other related genes. Among the gene mutations of HG-ESSs, BCOR shows the most common mutations in different ways. In current tumor therapies, immunotherapy is one of the most effective therapeutic approaches. In order to connect immunotherapy with HG-ESS, the understanding of tumor microenvironment (TME) is required. The TME of HG-ESS shows the mixture of tumor cells, vessels, immune cells and non-malignant stromal cells. Macrophages, neutrophils, dendritic cells and natural killer cells lose their expected functions, but rather show pro-tumoral functions by the matricellular proteins, extracellular matrix and other complicated environment in TME. In order to overcome the current therapeutic limitations of HG-ESS, immunotherapies should be considered in addition to the current surgical strategies. Checkpoint inhibitors, cytokine-based immunotherapies, immune cell therapies are good candidates to be considered as they show promising results in other stromal cancers and uterine cancers, while less studied because of the rarity of ESTs. Based on the advance of knowledge of immune therapies in HG-ESS, the new strategies can also be applied to the current therapies and also in other ESTs.

## Introduction

Endometrial stromal tumors (ESTs) are an uncommon, unique, and complicated subset of uterine mesenchymal cancers. ESTs show heterogeneous microscopic and genetic characters (1). The morphology of ESTs resembles normal proliferative endometrial stromal cells, so in most cases, an EST has to be identified by the genetic analysis and lesions (2).

ESTs can be classified into four groups along the criteria announced by the World Health Organization (WHO), i.e., endometrial stromal nodule (ESN), low-grade endometrial stromal sarcoma (LG-ESS), high-grade endometrial stromal sarcoma (HG-ESS), and undifferentiated uterine sarcoma (UUS). Molecular analysis of the tumor tissue is a promising method to classify ESTs. The number of members of UUS has been decreased as the technology of genetic analysis has been advanced. For example, NTRK-sarcomas were classified as a UUS, but this has been re-categorized as HG-ESS as the molecular mechanism has revealed (3).

The current therapeutic strategy of ESTs is surgical removal. For lesions limited in the uterus, en bloc removal of the affected and intact area is suggested. For HG-ESS patients with advanced-stage, adequate cytoreduction by metastasectomy is standard therapeutic protocol, while it is unclear that the cytoreduction improves the patient survival. Additionally, aggressive cytoreduction such as pelvic and para-aortic lymphadenectomy is not suggested with LG-ESS patients (4). Because the efficiency of adjuvant radiotherapy or chemotherapy is controversial, new medical strategies such as immunotherapy may have to be considered (5).

BCOR (BCL6 corepressor) gene resides on chromosome X, in the Xp11.4, and it has 16 alternative exons coding several proteins, with principal isoform encoded by 14 exons, giving rise to 1775 amino acids (6, 7). The nuclear protein of molecular mass ~190Kda is ubiquitously expressed in various tissues. However, the BCOR protein expression in adult human tissue is unknown (8). BCOR includes BCL-6-and MLLT3-binding domains, ANK repeats, and PUFD domain. The function of BCOR is mainly mediated by the BCL-6 binding domain, which interacts with the transcriptional repressor BCL-6, and the RAWUL domain, to which PCGF binds (7). BCOR genetic variation causes several carcinomas, and Gene fusions relating to it are associated with a diverse range of human neoplasms. BCOR mutation is directly related to cancer development by changing the protein’s usual RNA recognition preference by various alternation splicing at the pre-mRNA level (9, 10).

In recent findings, BCOR mutation induces HG-ESS in several clinical cases. BCOR ESS shows a broad range of clinical cases complicating the diagnosis and therapeutic strategies. We aim to enlighten the complicity of BCOR-ESS *via* the viewpoint of genetic alterations and the tumor microenvironment (TME) formations.

## HG-ESS

HG-ESS is a rare tumor officially recognized as a malignant tumor of the endometrial stroma in the 2014 WHO classification. HG-ESS is a stromal neoplasm displaying unclear uniform features intermediate between classic LG-ESS and UUS. They have characteristic genetic abnormalities t(10;17)(q22;P13), chiefly associated with the YWHAE-NUTM2 A/B fusion and often associated with a morphologically low-grade component. Morphological spectra vary according to genetic abnormalities. Recently, another subtype of a ZC3H7B-BCOR gene fusion-induced HG-ESS was discovered in new studies (11–14). Significant genetic alterations of HG-ESS show distinct characters and patterns histologically and mechanistically. The direct mechanistic evidence is not sufficient yet, but many of HG-ESS cases show the mutation of BCOR or other genes as the molecular analysis techniques advances (**Table 1**). YWHAE, NUTM2, EPC1, SUZ12, BCOR, BRD8, PHF1, ZC3H7B, TPR, NTRK1, LMNA, TPM3, RBPMS, NTRK3, EML4, COL1A1, PDGFB, STRN mutations are already reported mutations and other mutations may be revealed as the Next-generation Sequencing technique is being advanced.

**Table 1:** HG-ESSs with molecular alterations frequently reported.

Genes involved	Reported fusions/gene rearrangements/alterations	Translocations	References
*YWHAE*	*YWHAE/NUTM2*	t(10;17)(q22;p13)	(12)
*NUTM2A/B/E*	*EPC1-BCOR*	t(10;X)(p11;p11)	(15, 16)
*EPC1*	*EPC1-SUZ12*	t(10;17)(p11;q11)	(15, 16)
*SUZ12*	*BRD8-PHF1*	t(5;6)(q31;p21	(15, 16)
*BCOR*	*BCOR* alteration	none	(3)
*BRD8*			
*PHF1*			
*ZC3H7B*	*ZC3H7B-BCOR*	t(22;X)(q13;p11)	(14, 17)
*BCOR*	*BCOR-ZC3H7B*	t(X;22)(p11;q13)	
*BCOR*	*BCOR ITD*	none	(14)
*TPR*	*TPR-NTRK1*	1q31.1-1q23.1	(18)
*NTRK1*	*LMNA-NTRK1*	1q22-1q23.1	(18)
*LMNA*	*TPM3-NTRK1*	1q21.3-1q23.1	(18, 19)
*TPM3*	*RBPMS-NTRK3*	t(8;15)(p12;q25.3)	(18)
*RBPMS*	*EML4-NTRK3*	t(2;15)(p21;q25.3)	(19)
*NTRK3*	*COL1A1-PDGFB*	t(17;22)(q21.33;q13.1)	(18, 20)
*EML4*	*STRN-NTRK3*	t(2;15)(p22.2;q25.3)	(21)
*COL1A1*	*TPR-NTRK1*	1q31.1-1q23.1	(18)
*PDGFB*	*LMNA-NTRK1*	1q22-1q23.1	(18)
*STRN*	*TPM3-NTRK1*	1q21.3-1q23.1	(18, 19)

### YWHAE-NUTM2 Fusion

The YWHAE gene belongs to a broad family of proteins that mediate signaling by binding to phosphoserine-containing proteins (22). FAM22A/B was renamed NUTM2A/B due to sequence homology with NUT (NUTM1), famous for its role in nut midline carcinoma (22, 23). YWHAE-NUTM2 fusion tumors consisted of high-grade round and low-grade spindle cell components. The morphology is consisted of round cell sheets with intermediate size ovoid to round nuclei. Chromatin is open, and the staining pattern is scant to moderate eosinophilic cytoplasm. These sheets are adjacent to the fascicles of spindle cells resembling fibroblastic LG-ESS (16). Immunohistochemically, YWHAE-NUTM2 HG-ESS cells were perhaps immunoreactive to Cyclin D1 and BCOR.

### ZC3H7-BCOR Fusion

A retrospective molecular reanalysis of uterine sarcoma patients with BCOR gene rearrangements confirmed that ZC3H7B was the most common partner of gene rearrangement by fusion at either front or back of BCOR. In addition to ZC3H7B, ten other BCOR gene rearrangement partners have been identified, which are EP300-BCOR, BCOR-L3MBTL2, BCOR-RALGPS1, BCOR-NUTM2G, BCOR-MAP7D2, ING3-BCOR, RGAG1-BCOR, KMT2D-BCOR, BCOR-NUGGC, and CREBBP-BCOR (24). Molecular biologically, endometrial stromal sarcomas with BCOR rearrangement bear common MDM2 amplification and activation of the cyclin D1-CDK4 pathway by CDK4 amplification by cyclin D1 protein overexpression or CDKN2A deletion (25).

In tumors with BCOR gene rearrangements demonstrated sarcomas. Most cases were portrayed by spindle cells and various amounts of small round cell or epithelioid cell morphology. These cells usually have uniform nuclei, clear cytoplasm, and mild to moderate atypia. However, more minor cases exhibited moderate to severe atypia, illustrated by condensed chromatin, prominent nucleoli, and nuclear enlargement, often with an associated epithelioid or small round cell component. Additionally, some cases with BCOR rearrangements newly discovered sarcomas with epithelioid, spindle, or small round cell components and variable degrees of fascicular growth with collagenous or myxoid stromal change (24).

### BCOR Internal Tandem Duplication

HG-ESS often contains an oncogenic fusion, and however, some tumors have morphological overlap with HG-ESS even without gene fusion. This kind of genetic alteration is also found in some pediatric primitive sarcomas, including undifferentiated circular cell sarcoma and clear cell sarcoma of the kidney in infancy. This same subset of pediatric sarcomas lacks the oncogenic fusion but instead has an internal tandem duplication (ITD) associated with exon 15 of BCOR, the so-called BCOR ITD. According to previous studies, BCOR ITD was confirmed in 3 cases out of 26 HG-ESS, 2 cases were undifferentiated uterine sarcoma with uniform nuclear characteristics, and 1 case was diagnosed as YWHAE-NUTM2-negative HG-ESS. All three mutations have resulted in tandem replication of varying sizes of exon 15, which is the 3’ end of BCOR encoding the C-terminal end of BCOR protein (26).

### Other Alterations

EPC1-BCOR, EPC1-SUZ12, BRD8-PHF1, BCOR-ZC3H7 also show HG-ESS progression in the uterus (2, 15, 16). As a newer type of HG-ESS is being discovered, histopathological observation is not enough to identify a specific type of genetic alteration. This is why genetic analysis of ESS samples is required to distinguish the kind of ESS, including HG-ESS, such as the next-generation sequencing or even Sanger sequencing of the RT-PCR product (**Table 1**).

## Tumor Microenvironment of Sarcomas

**Figure 1:**
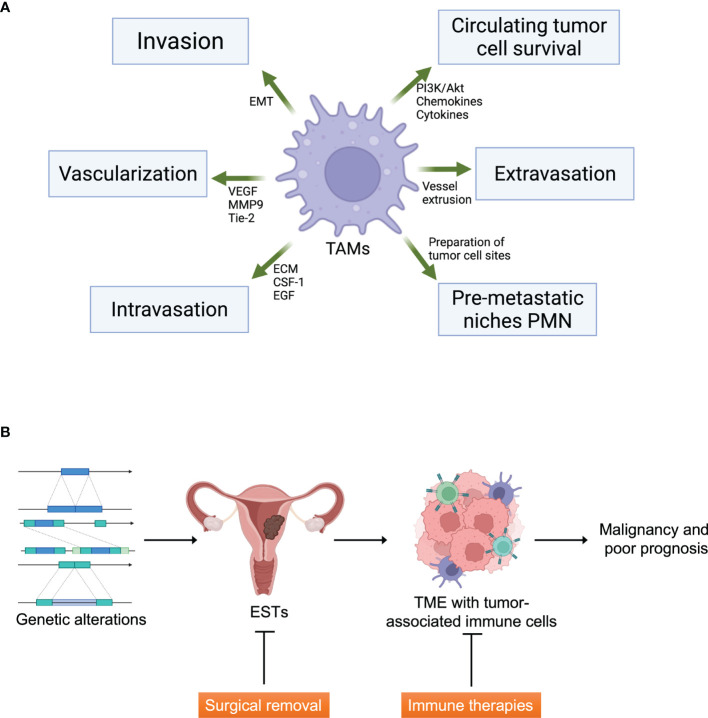
Tumor microenvironment and ESTs. **(A)** The functions of tumor-associated macrophages (TAMs) in tumor microenvironment formation. TAMs affect tumor cell metastasis including invasion, vascularization, intravasation, formation of pre-metastatic niches and protection of circulating tumor cells. EMT, epithelial-mesenchymal transition; VEGF, vascular endothelial growth factor; MMP9, matrix metallopeptidase-9; ECM, extracellular matrix; CSF-1, colony-stimulating factor-1; EGF, epithelial growth factor. Created with Biorender.com. **(B)** The graphical abstract of ESTs and therapeutic strategies. Immune therapies can be an additive strategy to cure malignant ESTs targeting TME. Created with Biorender.com.

The tumor microenvironment (TME) of stromal sarcomas comprises non-malignant stromal cells, blood vessels, immune cells, and tumor cells. Matricellular proteins and extracellular matrix (ECM) proteins arising from stromal cells are crucial for cellular movement *via* structural support and signal transduction. Immune cells in TME of sarcomas are composed of innate immune cells such as neutrophils, tumor-associated macrophages (TAM), tumor-associated dendritic cells (TADCs), and natural killer (NK) cells, and adaptive immune cells like B-cells and T-cells. TAMs, TANs and TADCs show protumoral functions leading to metastasis and cell invasion, ECM remodeling and angiogenesis by suppressing immune surveillance for antitumoral effect (27). TAMs share much of the characters of M2 macrophages, which is protumoral. As EST turns malignant, M2 is being dominant over M1 TAMs. Dominant M2 portion in malignancy is because angiogenic environment such as VEGF, hypoxia and epigenetic derangements (28). TANs are mostly consisted with protumoral N2 cells rather than antitumoral N1 cells like the case of TAMs. TADCs lose the antigen-presenting cell (APC) function and obtain protumoral effect (29). The composition of individual subsets of these immune cells differs significantly depending on the prior treatment, primary tumor location, sarcoma subtype, and genetic background. TAM is one of the four major subgroups of tumor-associated myeloid cells (TAMCs), which also include tumor-associated neutrophils (TANs), myeloid-derived suppressor cells (MDSCs), and Tie2-expressing monocytes (TEMs) (**Figure 1A**) (30–32). Several activated and antigen-specific T-cell therapies have been tested for sarcomas, which showed exhaustion of T-cells by immune regulation by TME such as mesenchymal stem cells (MSCs), regulatory T cells (Tregs), and TAMs.

The strong correlation of macrophages and sarcomas shows TAMs may have to be therapeutically targeted to overcome tumor progression and patient survival in sarcoma patients (33–36). Even though TAMs phagocytose necrotic tumor cells, TAMs show tumor-promoting functions and immunosuppression in sarcomas. For example, TAMs increased number of TAMs in TME induced the decrease of the effect of chimeric antigen receptor (CAR) T-cell immunotherapy (37). Meanwhile, the entire mechanism of the number and density of TAMs in sarcomas should still be investigated to promote tumor progression and immune cell profiles.

## Immune Suppression in Endometrial Cancers

CIBERSORT is a retrospective in silico analysis. CIBERSORT enables profiling immune cells through the deconvolution of gene microarray data sets (35, 38). The deconvolution reconstructed relative quantity, and the immune cell subsets residing in tumor tissue. The cell-type determination is made from the gene expression dataset by matching the information of 547 markers of 22 known peripheral immune cells (38). This method has the advantage of enabling detecting functionally distinct and rare immune cell types such as Tregs, γδ T cells, mast cells, and memory B cells (35). Flow cytometry has effectively confirmed this advanced technology and is used to decide the composition of infiltrated immune cells in many different malignant tumors like colon cancer and breast cancer (39, 40).

The immunological aspect of the endometrial TME has been less studied, unlike ovarian and other solid tumors. Furthermore, ESS has been even less studied than endometrial adenocarcinomas. In this aspect, it is worthwhile to learn the TME of adenocarcinomas to apply to ESSs. Especially TAMs and antitumor adaptive immune responses, FoxP3^+^ Tregs in endometrial cancers is not the only issue of adenocarcinomas as they are the most abundant immune cells in stroma (41).

## Possible Immunotherapeutic Strategies

The possible involvement of the immune system in regulating cancer was first observed in patients with sarcoma when Wilhelm Busch of Germany reported tumor regression in 1866 of a sarcoma patient who developed an erysipelas infection (42). Immunotherapy to treat sarcoma can be traced back to at least 1891. At the time, William Coley, a noticeable orthopedic surgeon at New York Memorial Hospital, currently Memorial Sloan Kettering Cancer Center, developed what was known as “Coley’s Toxin” to treat a series of osteosarcomas (43, 44). He found that injection of a streptococcal organism (originally a live bacterium, a mixture that was later killed by heat containing Serratia marcescens) could induce remission in some patients with inoperable sarcoma. His use of the toxin was controversial and eventually lost popularity, but many consider it today as a forerunner of modern anticancer immunotherapy (45).

Maybe the best definition of modern immunotherapy is from Paul Ehrlich’s early 1900’s description of “Magic bullet” - a specific drug that only attacks and kills diseased cells, leaving no surrounding normal cells (46). The increased frequency of lymphoid malignancies in immunocompromised patients suggests that the immune system plays an essential role in carcinogenesis (47). In addition, the development of sarcoma is well known in allograft recipients, and the risk of developing it in non-immunocompromised patients more than doubles (48). As the cancer treatment adapts personalized and tailored medicine, personally tailored immunotherapies may be an additional plus to the traditional immunotherapeutic strategies. In this section, we want to mention currently available immunotherapies that may treat HG-ESS.

Targets of immune checkpoint inhibitors include programmed cell death protein-1 (PD1), and its ligands, PDL1/PDL2, mucin protein 3 (Tim3), and its ligand galectin-9, T cell immunoglobulin, and CTL-associated antigen 4 (CTLA-4). Blocking CTLA-4 while priming has been reported to the CD8+T cell accumulation leading to the production of effector cytokines like INF-γ. For example, CTLA-4 blockade successfully showed decreased tumor size in the mouse stromal cancer model (49). Numbers of tumoral CD4+ and CD8+ T-cells or CD8/Treg ratio in tumors are not affected by CTLA-4 blockade. Still, the increased production of IFN-γ from tumoral CD8+ T-cells shows the benefit of CTLA-4 inhibition (50).

PD1 is exclusively expressed in immune cells such as T-cells. Meanwhile, PD-L1 is widely expressed by different cells, especially tumor cells. The interaction of PD1 and PD-L1 is one of the significant immune escape mechanisms of tumor cells. PD-L1 mRNA expression is highly upregulated in stromal cancer cells as well as heterogeneously expressed across tumor tissue (51). One confusing point of PD-L1 is that patients with low PD-L1 expression may have higher metastatic risk than patients with high PD-L1 expression. The analysis of immune checkpoint molecules from stromal cancers found PD1, lymphocyte activation gene 3 (LAG3), and T-cell immunoglobulin mucin 3 (TIM3) upregulation on tumor-infiltrating T-cells compared matched control blood cells (52). Although PD-L1 expression in ESTs has not been studied systemically, PD-L1 has been positively expressed in primary tumors of 77% patients and 30-40% in metastatic lesions from 88 cases of uterine cancer (53). Although this report revealed PD-L1 or PD-1 expression is not directly associated with the prognosis, the origin of cells was not categorized, so further detail study from EST patients is required. Considering the prognostic value of PD-L1 in other stromal cancers, the importance of analyses in ESTs seems to be required.

The next considered immune checkpoint in stromal cancers are TIM3 and its binding partner, galectin-9. TIM3 is expressed on immune cells, while galectin-9 is expressed on cancer cells. The pathway of TIM3 and galectin-9 has been known for their role in cancer malignancies. Blocking this pathway is being actively investigated in the meaning of immune checkpoint inhibition (54, 55). Studies on TIM3 revealed NK cells and tumor tissues show the expression of TIM3 and galectin-9, respectively (55, 56). This shows TIM3/galectin-9 may also have an essential role in HG-ESS, even though the direct evidence is low. Currently, Nivolumab which inhibits PD-1 is under clinical trials with or without the combination of Ipilimumab, which inhibits CTLA-4 (57). It is not easy to expect how the outcome of the trials will be, but immune checkpoint inhibitors can be an excellent candidate in HG-ESS therapy in the future.

Cytokine-based immunotherapy can be the following strategy for HG-ESS. Type I interferons such as IFNα are a physiological danger signal that promotes Th1 responses and memory T-cell differentiation. IFNα treatment combined with imatinib remarkably achieved complete responses in stromal cancer patients (58). IFNγ is well-known for its innate and adaptive immunity role and is produced by activated NK cells, NKT cells, CD4+ T-cells, and CD8+ T-cells. IFNγ was decreased in stromal cancer cell patients, but IFNγ producing cell subsets increased significantly after IFNα treatment. Furthermore, tumor-infiltrating leukocytes (TILs) of the patients actively expressed IFNγ after IFNα treatment (58).

Immune cell therapy is a highly focused area in cancer therapy. For example, the chimeric antigen receptor (CAR) T-cells are genetically modified specific cancer-specific T lymphocytes produced against a particular tumor antigen of a specific cancer cell. T-cells are isolated from a patient’s peripheral blood and then activated. Retroviral or lentiviral transduction with a CAR lets the T-cell express the surface receptor, explicitly recognizing the tumor cells with the cognate antigen. Several studies observed CAR-T-cells are effective in stromal cancers and uterine cancers, which is also a promising therapeutic future of HG-ESS (59, 60). These results show that CAR T-cells have a substantial role in the immunotherapy against stromal cancers, including ESS. Additionally, a recent finding shows active neutrophils drive unconventional T cells to mediate resistance to sarcomas in mouse and some human cancer (61). With the help of neutrophils, T cells are polarized to UTCαβ and type 1 immunity is strongly activated, which may lead to the resistance to the stromal tumors including HG-ESS and other ESTs.

The strategies stated in this section, such as checkpoint inhibitors, cytokine therapy, and immune cell therapy, are not being vigorously studied for the treatment of HG-ESS currently. However, the potential of immunotherapy is highly promising considering the successful results from the other stromal cell tumors and uterine cancers. The need of promising survival of patients still exists with advanced HG-ESS patients with cytoreduction surgery. Because the complete removal of the tumor is not available in patients with advanced cancer, uncertain radiotherapy and chemotherapy have been the only options for those patients. So, the development of immune therapy will be the next therapeutic option for patients with advanced HG-ESS.

## Conclusions

Recent advances in Next Generation Sequencing (NGS) have made tremendous efforts to incorporate the immunohistochemical and morphological EST classifications. This new change enabled molecular subclassifications, making HG-ESS identification clearer than before. The purpose of the molecular classification of ESTs is to understand the molecular biology to the development of specific-targeted therapies for each subcategory. This is very important for planning treatment strategies for metastatic disease. Therefore, we will observe the discovery of new individuals and gradually replace histopathological classification with molecular classification based on genetic analysis.

Scientific and medical evidence highly supports the perception that host immunity can be suppressed in many different mechanisms to eradicate and control stromal cancers. It is always complicated to fight rare cancers with various genetic mutations. However, new results configure the actions, types, and prognostic significance of TILs and clarify the mechanisms. TILs can be handled directly or in combination with other molecular therapeutic strategies to optimize tumor cell death. However, limiting the toxicity of this strategy should be considered. Immunotherapies are still in very early steps in experiments and development, but their potential in cancer therapy is tremendous and must be explored robustly to cure patients with ESS.

HG-ESS patients with advanced-stage have few therapeutic options when the tumor cannot be surgically removed ideally. However, adjuvant radiotherapy and chemotherapy have a prominent disadvantage, which shows inconsistent and controversial therapeutic efficiency. Personalized and tailored immunotherapy such as cytokine therapies, immune checkpoint inhibitors, and immune cell therapies remarkably succeeded in several advanced cancers, including stromal cancers and uterine cancers. This is why immune therapy should be considered in HG-ESS patients. Furthermore, based on the advance of knowledge of immune therapies in HG-ESS, the new strategies can also be applied to en bloc resection-available HG-ESS, LG-ESS, and UUS in the future (**Figure 1B**).

## Author Contributions

YK and DK wrote the original draft. WJS and JH supervised and finished the manuscript. All authors contributed to the article and approved the submitted version.

## Funding

JH was supported by research grants from Daegu Catholic University in 2021 (20213022).

## Conflict of Interest

The authors declare that the research was conducted in the absence of any commercial or financial relationships that could be construed as a potential conflict of interest.

## Publisher’s Note

All claims expressed in this article are solely those of the authors and do not necessarily represent those of their affiliated organizations, or those of the publisher, the editors and the reviewers. Any product that may be evaluated in this article, or claim that may be made by its manufacturer, is not guaranteed or endorsed by the publisher.
